# The Non-Surgical Treatment of Suppuration

**Published:** 1898-03

**Authors:** 


### THE NON-SURGICAL TREATMENT OF SUPPURATION.

In a recent article by the gifted author of "Syphilis in the Innocent," L. Duncan Buckley, A. M., M. D., which appears in the *British Medical Journal*,* there is a fund of sound sense and good reasoning. It appears strange and no less charming to see the leaders of the profession thus arraying their cohorts of scientific and bacteriologic study along the lines of homely practice of the country doctor. Yet true it ever is that many of the ways of the country doctor are correct absolutely without his knowing the scientific reason why. We say "the country doctor" and we mean the old time country doctor, for to-day our country men are the pride of our polyclinic schools and equal in their analytic power and their knowledge of pathological processes most of their colleagues whose surroundings give them much greater facilities for scientific advancement.

In the article referred to, Dr. Buckley tells us of the non-surgical treatments of boils, carbuncles and felons. It has been so long the custom to treat these cases purely from the standpoint of the pathologist and attack the invading germ, losing sight of the patient in the deal, that the doctor does well thus to call cur attention to the line of defense that nature supplies for us. The key-note of the doctor's article is "*build up*, get the patient in perfect condition" and his position is a good one. He says:

"Granted the pyogenous powers of the micro-organisms, my position is that their powers are temporary, and limited by their own nature and the condition of the tissues in which they are found. Witness the different results attending superficial wounds in different individuals; and also in the same individual at different times. In some persons a scratch or bruise will readily take on suppurative action, while in another it tends to heal in the kindest manner possible, with lit-

*The Non-Surgical Treatment of Boils, Carbuncles, and Felons by L. Duncan Buckley A. M., M. D., *British Medical Journal*, October 2, 1897.

tle or no pus. The same is seen in the same patient at different times, and there is certainly some measure of truth in the common remark that 'one's blood must be in a bad condition,' because of the occurrence of suppurative areas with slight injury. I am confident that in my own person there is a constant difference in this regard, from time to time. The same may be observed constantly in practice in connection with eczema and many skin lesions. Thus, although the pus bacili are undoubtedly everywhere present and at all times ready to lodge and do their work if the soil is proper, yet in comparatively few instances is suppuration set up. This is only in accordance with what is continually observed in the prodigality of nature, where, of the masses of pollen produced only an exceedingly small proportion ever fructifies; and of the millions of seeds developed relatively few reproduce the species, and these only when the soil and other conditions are favorable.

"I may be pardoned for thus dwelling on what is tacitly admitted on all sides, perhaps by every one, theoretically, for, practically, it seems to be forgotten or overlooked by many in the treatment of the conditions which form the subject of this paper. While it is continually granted that attention should be paid to the general condition of patients with these affections, I find that as a matter of fact very little is generally done. There is commonly so much regard given to the local trouble that there is not the careful investigation of the general condition and direction of the patient's life which will lead to the raising of the vital forces above the pus-producing stage. The yielding of one or more points to the attacks of the pus parasite too often attracts attention from the weakened system and debilitated tissues, which may allow of many more successful attacks, as commonly happens in the case of boils.

"Iron is most commonly needed, but quite as often there will be digestive and assimilative difficulties also to be overcome. Sometimes the cause lies only in overwork and worry, often in dissipation, though of a relatively harmless kind, involving late hours and irregular eating. I can not help dwelling as strongly as possible on this constitutional aspect of the question, for in this I find myself at variance with much that is taught even in the best modern text-books. In them this aspect appears to be regarded apparently as of relatively minor importance, compared to the local treatment, whereas I regard it as of the first importance, as compared to the relatively simple local treatment, about to be described, which I have found thoroughly satisfactory.

"The combination of iron which I have most commonly used in the affections is one which is known to us as Startin's mixture, somewhat according to the following formula:

℞.—Ferri sulphatis, ʒj.

Magnesiæ sulphatis, ʒvj.

Acidi sulphurici dil, ʒiv.

Sy.zinziberis, ʒiv.

Aquæ, ad ℥iij.

M.—Teaspoonful in water, through a tube, after meals.

"Unless there is some counter indication I generally begin treatment also with a good mercurial purge: R.—Massæ hydrargyri, Extract. Colocynth. co. aa gr. x, Pulv. ipecac gr. ii, M. Div. in pil. No. iv. Take two at night and and two on the second night after. These four pills are generally repeated at the end of a week, and perhaps in other successive weeks.

"Sulphide of calcium, if a perfectly good and fresh article, and properly used, has, in my experience, a very decided controlling effect over the process of suppuration. I always give it in gelatin coated pills, which I test myself, for occasionally the drug will be found quite ineffective, from having changed to the sulphate of lime or gypsum. To be efficient it should be given freely, one-quarter grain every two hours, say eight or ten doses during the day; this in connection with the iron tonic."

To this treatment he adds the following ointment thickly spread upon cotton and gently held in place by strips of adhesive plaster placed above and below the boil:

℞.—Acidi carbolic, gr. v–x.

Extr. ergotæ, fl'd ʒi-ʒij.

Pulvis amyli, ʒij.

Zinci oxidi, ʒij.

Ungent. aquæ rosæ, ℥ij.

M.—et ft. unguent.

—*Kansas City Medical Index.*

